# Development of a Toluene Detector Based on Deep UV Absorption Spectrophotometry Using Glass and Aluminum Capillary Tube Gas Cells with a LED Source

**DOI:** 10.3390/mi10030193

**Published:** 2019-03-18

**Authors:** Sulaiman Khan, David Newport, Stéphane Le Calvé

**Affiliations:** 1 School of Engineering, Bernal Institute, University of Limerick, V94 T9PX Limerick, Ireland; sulaiman.khan@ul.ie (S.K.); david.newport@ul.ie (D.N.); 2 Université de Strasbourg, CNRS, ICPEES UMR 7515, F-67000 Strasbourg, France; 3 In’Air Solutions, 67087 Strasbourg, France

**Keywords:** ultraviolet light-emitting diode (UV LED), spectrophotometry, UV absorption, gas sensors, Benzene, toluene, ethylbenzene and xylene (BTEX), toluene, hollow core waveguides, capillary tubes

## Abstract

A simple deep-ultraviolet (UV) absorption spectrophotometer based on ultraviolet light-emitting diode (UV LED) was developed for the detection of air-borne toluene with a good sensitivity. A fiber-coupled deep UV-LED was employed as a light source, and a spectrometer was used as a detector with a gas cell in between. 3D printed opto-fluidics connectors were designed to integrate the gas flow with UV light. Two types of hollow core waveguides (HCW) were tested as gas cells: a glass capillary tube with aluminum-coated inner walls and an aluminum capillary tube. The setup was tested for different toluene concentrations (10–100 ppm), and a linear relationship was observed with sensitivities of 0.20 mA·U/ppm and 0.32 mA·U/ppm for the glass and aluminum HCWs, respectively. The corresponding limits of detection were found to be 8.1 ppm and 12.4 ppm, respectively.

## 1. Introduction

Monitoring of air quality in indoor spaces is critical for healthy living. Nowadays most of our daily activities are based in indoor spaces where exposure to various indoor air pollutants is inevitable [1]. Indoor air can contain various volatile organic compounds (VOCs) among other pollutants such as air-borne particles, microorganisms, household odours, and gases. VOCs are organic compounds with a high vapour pressure at room temperature; they readily evaporate into a gaseous phase at room temperature. Some of the common VOCs are acetaldehyde, acetone, benzene, carbon tetra chloride, ethyl acetate, heptane, hexane, isopropyl alcohol, formaldehyde, naphthalene, styrene, toluene, and xylenes [1,2]. Benzene, toluene, ethylbenzene and xylene (BTEX) are aromatic hydrocarbons and are some of the most hazardous pollutants among VOCs. Toluene is a colourless VOC with a sweet, pungent odour, density of 0.866 g·cm^−3^ at 20 °C, and boiling point of 110.7 °C [3]. Its sources of generation in indoor spaces are common household items, i.e., cleaning products, paint thinners, adhesives, synthetic fragrances, nail polish, and cigarette smoke. Automobile emissions are the main source of toluene in outdoor air environments [4]. Exposure to toluene can affect the central nervous system, liver, kidney, and skin [3]. The American Conference of Governmental Industrial Hygienists (ACGIH) have established the threshold limit value (TLV) of 50 ppm for toluene for 8 h exposure. In addition, the Occupational Safety and Health Administration (OSHA) recommends permissible exposure limits (PEL) for toluene of 200 ppm as the 8 h time-weighted average (TWA) concentration [5].

Detection of aromatics VOCs at ppm and sub-ppm ranges requires a sensitive and accurate method. Different techniques have been applied for the detection of different VOCs, for instance, electrochemical gas sensors [6], micro gas chromatography (µ-GC) [7], photoionization detectors [8], piezoelectric-based gas sensors, i.e., surface acoustic wave [9], quartz crystal microbalances [10], and tuning forks [11], gravimetric-based gas sensors [12], metal-oxide semiconductor gas sensors [13], and optical sensors such as colorimetric gas sensors [14], non-dispersive infrared gas sensors [11], and ultraviolet (UV) spectrophotometry gas sensors [15]. Among these, optical gas sensors are highly sensitive, they have minimal drift issues and rapid time responses, and they facilitate real time and in situ measurements without changing the chemical nature of gases.

UV spectrophotometry is a non-destructive, rapid time response with minimal cross-responses to other gases as long as its design is carefully considered. The technique involves direct measurement of a molecular absorption at a specific wavelength which offers an inherently reliable approach for gas sensing with excellent selectivity [16]. BTEX gases absorb strongly in the deep UV range, i.e., 250–270 nm [17], indicating they can be detected using deep UV spectrophotometry. 

Recently, deep ultraviolet-light-emitting diodes (UV-LEDs) with narrow bandwidths (<30 nm) have been developed, which matches with the absorption band of many molecules. It provides a portable source without the need of monochromators or filters. LEDs have good stability, robustness, flexibility in output intensity, low power consumption and low heat generation [18]. In order to broaden the emission band, an array of LEDs of different wavelength can be used to cover a broad range of molecules [19]. UV-LEDs have been applied in the detection of different gases, for instance, NO_2_ [20], O_3_ [21], SO_2_ [22], and BTEX [23].

In this work, we have applied UV spectrophotometry to detect toluene using a fiber-coupled deep UV-LED with aluminium- based hollow core waveguides (HCWs) coupled to mini spectrometer. We have assembled the fluid and optic parts using 3D connectors, which makes the alignment and sealing of the setup easier.

## 2. Materials and Methods

### 2.1. Spectrophotometry

UV absorption spectrophotometry is a direct optical gas detection method that is based on the unique absorption spectra (fingerprints) at a specific wavelength. In spectrophotometry, the molecular absorption level is measured according to the Beer–Lambert law:

(1) $$A=\sigma cl=log\frac{I_{0}}{I}$$

where *A* is absorbance, *σ* (cm^2^/molecule) is the absorption cross-section, *c* (molecules/cm^3^) is concentration of gas molecules, and *l* (cm) is length of gas cell. *I*_0_ and *I* are the transmitted intensities recorded for the background gas (i.e., nitrogen) and toluene gas concentrations in the gas cell, respectively. The constant *σ* is the absorption cross-section, which is a molecule-specific property and has a constant value at a specific wavelength. It represents the effective area of a molecule that is needed for a photon to transverse the molecule.

The sensitivity depends on the optical path length, which is defined by the design of the absorption gas cell. The absorption cell can be single pass, multi-pass, or a resonant cavity. The single pass cell is relatively easy to manufacture, has a quick time response and is relatively easy to couple with light sources and detectors compared to multi-pass or resonant cells. HCW offer a compact and efficient alternative to gas cells. They provide a compact platform for the interaction of photons and gas molecules to realize a quantitative and molecular specific absorption spectroscopy. The HCWs guide the radiation in a leaky-mode and the radiation is propagated by metallic reflection inside the coaxial hollow core. The transmission of UV in HCW depends on material, size and geometry of HCW. HCW with smaller diameter have higher attenuation losses compared to the higher diameter. They have been applied in IR spectrometry and other sensing applications, e.g., biomedical and toxicology.

### 2.2. Instrumentation

A fiber-coupled deep UV-LED (Mightex Systems, Pleasanton, CA, USA) with a peak at 260 nm and power range from 45–80 µW was used. A mini-spectrometer (Hamamatsu mini-spectrometer C10082CH, Iwata, Japan) with a spectral detection range from 200–800 nm and integration time of 10–10,000 ms was used to record the intensity of the transmitted UV light. Two HCWs: one glass capillary tube with an inner wall coated with aluminium (Doku Engineering, Japan) and the other aluminium capillary tube as a waveguide (Advent, London, UK), were tested. The gas cell was coupled with the source and detector using optical fibers (Ocean Optics, Largo, FL, USA) for UV applications (range 200–1100 nm) with a core diameter of 400 µm. The optics and fluidics connections were 3D printed. The connectors were designed using Solidworks 2018 and 3D printed (Ultimaker 3, Geldermalsen, The Netherlands) using Acrylonitrile Butadiene Styrene (ABS). Toluene was delivered from a gas cylinder with concentration of 100 ppm ± 2% (Air Products, Aubervilliers, France). The gaseous flow was controlled via two mass flow controllers (Bronkhorst, Gelderland, The Netherlands) with a full-scale range of 20 mL/min ± 0.5% and 50 mL/min ± 0.5%.

### 2.3. Experimental Setup

**Figure 1:**
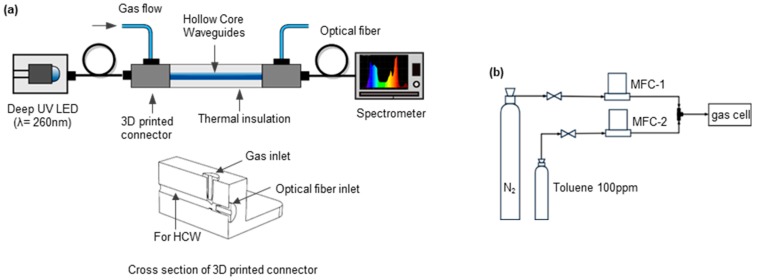
(**a**) Experimental setup for toluene detection. (**b**) Toluene concentration generation setup.

The schematic of experimental setup is shown in Figure 1a. In order to ensure a stable intensity, a constant current was supplied to the LED and the emission intensity was varied by changing the input current (0–30 mA). The opto-mechanical components were aligned using 3D printed holders on the optical breadboard to avoid baseline shifts due to mechanical movement. The gas cell was thermally insulated to minimize thermal fluctuations.

Different concentrations of toluene were generated in nitrogen using a configuration of mass flow controller (MFC) as shown in Figure 1b. The desired concentration of toluene was obtained by mixing toluene with nitrogen using MFC-2 and MFC-1, respectively. A total gas flow of 40 mL/min was injected into the waveguide gas cell. The sealing of the opto-fluidics connector was tested, and less than 3% leakage was found. 

## 3. Results and Discussion

The glass HCW and aluminium HCW were investigated for gas sensing applications. The glass HCW was composed of a glass capillary tube with a thin inner coating of aluminium. The aluminium HCW was a capillary tube made of tempered aluminium with 99.7% purity. The lengths of the glass HCW (inner diameter, 1mm; internal gas cell volume, 27 µL) and aluminium HCW (inner dimeter, 2 mm; internal gas cell volume, 157 µL) installed were 34 cm and 50 cm, respectively. Aluminium was selected because it had good reflective and transmittance properties (i.e., attenuation loss, 0.2 dB/m) for deep UV application, compared to other reflective metals like silver [24]. It also has good chemical compatibility with toluene.

**Figure 2:**
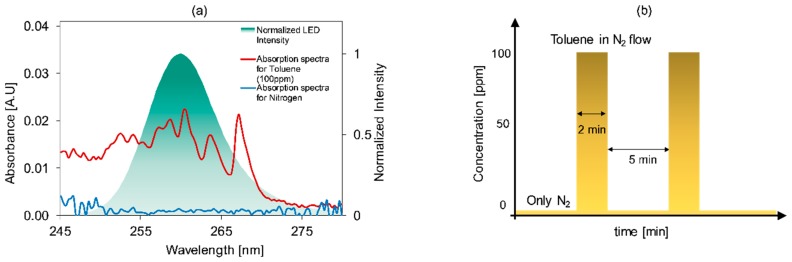
(**a**) Absorption spectra of toluene (100 ppm), background gas, i.e., nitrogen, and emission spectrum of deep UV-LED. (**b**) Schematics of flushing routine of nitrogen and toluene for measuring reference and measured intensity respectively.

The absorption spectra of toluene and the emission band of UV-LED was measured and compared, as shown in Figure 2. Toluene had three peaks for a wavelength range of 250 to 270 nm. The bandwidth (full width at half maximum (FWHM)) of the LED is 10 nm, centred at 260 nm, covering the absorption spectra of toluene, which implies that the setup can be applied for measuring the absorbance of toluene.

The measurement sequence was started by flushing nitrogen through the gas cell, followed by injecting different concentrations of toluene for 1 to 2 min until a stable signal was obtained as shown in Figure 2b. The recorded intensities for nitrogen and toluene concentrations were used as a reference and measured intensity, respectively. A dark intensity was recorded and subtracted from both references, i.e., *I*_0_ and the measured *I* intensities to calculate the absorbance according to Equation (1).

**Figure 3:**
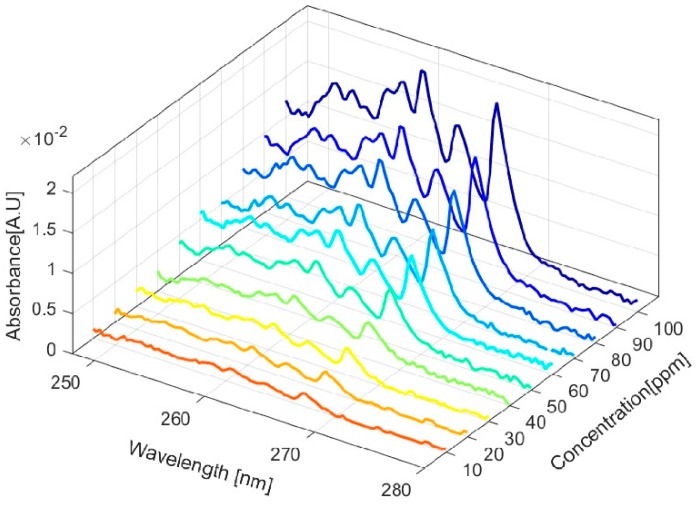
Absorbance of toluene at different wavelengths for different concentrations of toluene using the glass hollow core waveguide (HCW).

The setup with the glass HCW was tested for a concentration range of 10–100 ppm. The absorbance increased linearly with an increasing toluene concentration according to the Beer–Lambert law, as shown in Figure 3.

**Figure 4:**
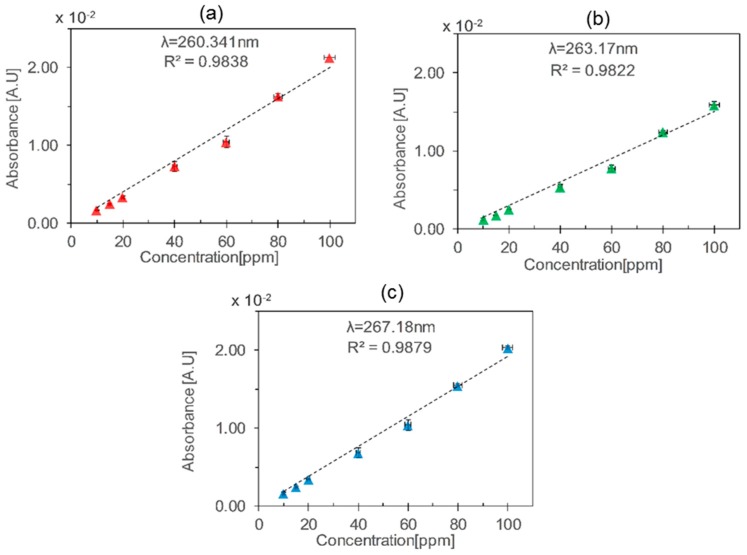
Absorbance vs. toluene concentration using the glass HCW at different wavelengths: (**a**) λ_1_ = 260.34 nm, (**b**) λ_2_ = 263.17 nm, (**c**) λ_3_ = 267.18 nm. The vertical and the horizontal error bars represent standard deviations in absorbance values and combined uncertainties in the generated concentrations defined by the uncertainties of mass flow controllers (MFCs) and gas cylinder concentrations, respectively.

The absorbance peaks at λ_1_ = 260.3 nm, λ_2_ = 263.1 nm, and λ_3_ = 267.2 nm were plotted for different concentrations of toluene, and a linear relation was obtained, as shown in Figure 4. The combined uncertainties in the toluene concentration were calculated according to BIPM guidelines [25], by taking into account the uncertainties of MFCs and gas cylinders. The sensitivity was calculated by taking the slope of the plot, representing absorbance vs. concentration. Sensitivities of 0.20 mA·U/ppm, 0.15 mA·U/ppm, and 0.19 mA·U/ppm were obtained for λ_1_, λ_2_, and λ_3_ respectively. According to the absorption cross-section values at λ_1_, λ_2_, and λ_3_, the absorbance values should be higher for λ_3_, but relative lower values were observed due to the emission profile of LED and limited resolution of spectrometer. A limit of detection of 8.2 ppm was calculated for λ_1_ from standard deviation of the calibration data assuming that the data is normally (Gaussian) distributed, using the equations [26].

(2) $$x_{\mathrm{LOD}}=\;\frac{S_{y}t}{r}$$

*x*_LOD_ is the limit of detection, where *r* and *S_y_* represent sensitivity of the linear fit and average standard deviation respectively.

(3) $$r=\frac{\Delta y}{\Delta x}=\;\frac{n\;\sum \left(x_{i}y_{i}\right)-\;\sum x_{i}\;\sum y_{i}}{D}$$

(4) $$S_{y}=\;\sqrt{\frac{\sum {\left(y_{i}-r\;x_{i}-b\right)}^{2}\;}{n-2}}$$

where *b* is intercept of the calibration curve i.e. the signal offset.

(5) $$b=\;\frac{n\;\sum x_{i}^{2}\;\sum y_{i}-\sum \left(x_{i}\;y_{i}\;\right)\;\sum x_{i}}{D}$$

(6) $$D=n\sum x_{i}^{2}-{\left(\sum x_{i}\right)}^{2}$$

where *t* is Student t-function. *x_i_* and *y_i_* are the calibration curve points and *n* is the number of data points on calibration curve.

**Figure 5:**
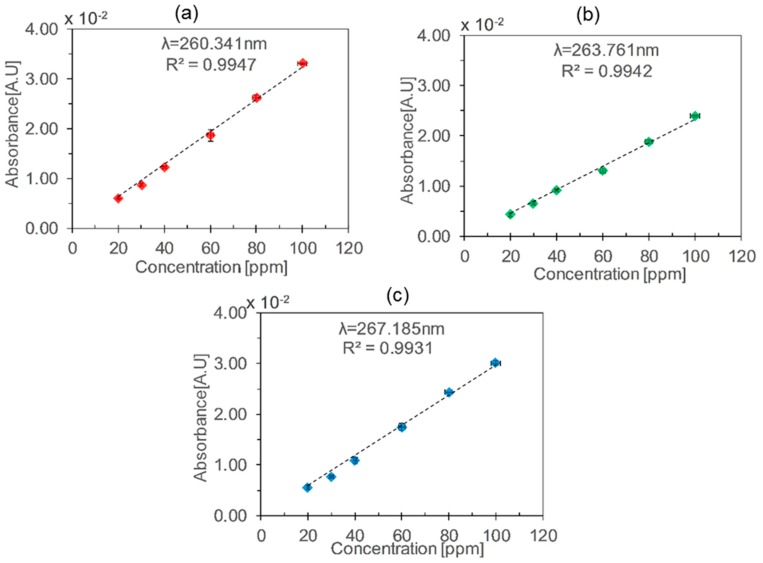
Absorbance vs. toluene concentration using the aluminium HCW at different wavelengths: (**a**) λ_1_ = 260.34 nm, (**b**) λ_2_ = 263.17 nm, and (**c**) λ_3_ = 267.18 nm. The vertical and the horizontal error bars represent standard deviations in absorbance values and combined uncertainties in the generated concentrations defined by the uncertainties of MFCs and gas cylinders, respectively.

The same experiment was repeated for the aluminium HCW and a good linearity was found for different concentrations of toluene (20–100 ppm) as shown in Figure 5. Sensitivities of 0.32 mA·U/ppm, 0.23 mA·U/ppm and 0.30 mA·U/ppm were obtained for λ_1_, λ_2_ and λ_3_ respectively. A limit of detection of 12.5 ppm was calculated for λ_1_.

**Figure 6:**
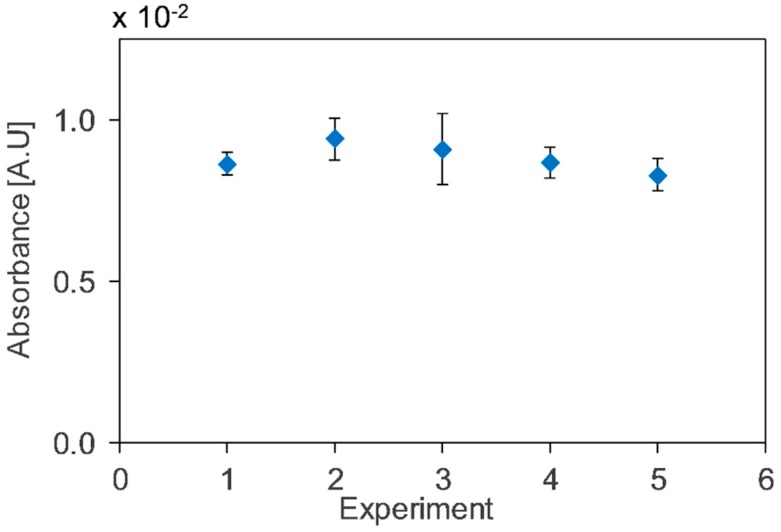
Absorbance for different experimental conditions for repeatability and reproducibility. (1) flowrate, 40 mL/min; (2) flowrate, 20 mL/min; (3) flowrate, 10 mL/min; (4) Detector integration time, 50 ms; and (5) LED current input, 15 mA.

The repeatability and reproducibility of the system were tested for a toluene concentration of 30 ppm using aluminium HCW by varying the gas cell flow rate, LED current input, and the integration time of the spectrometer. A good repeatability (relative standard deviation, RSD = 2.5%) was found for five experiments using a flow rate of 20 mL/min with LED current input of 30 mA and integration time of 100 ms (experiment 2 in Figure 6). Reproducibility with RSD = 4.8% was observed for five different experiments at different conditions, as shown in Figure 6.

The performance of the two HCWs for toluene detection applications is compared and summarized in Table 1. The aluminium HCW had a higher sensitivity compared to the glass HCW at the different peak wavelengths. Also, the aluminium HCW had good mechanical properties and can be easily assembled with fluidic and optical components. There were low coupling optical losses associated with the aluminium HCW, resulting in improved sensitivity. On the other hand, the glass HCW was fragile, and it was challenging to obtain a smooth end surface for alignment with the LED source and detector. The aluminium HCW offered a simple, cost-effective, and robust approach for absorption spectrometry with a good level of sensitivity. On the other hand, the glass HCW was a good candidate for application where a low gas cell volume is needed, for example integration of the detector with a μ-GC.

By comparing the performance of the sensor with other toluene sensing methods, the UV absorption detector based on HCW had a good sensitivity and linearity at room temperature. For instance, resistive gas sensors have a linearity range between 10–100 ppm, but they operate at high temperature [13]. Fiber optic toluene sensors have limited linearity and selectivity. Optical fiber sensors based on a long period grating for toluene has an operating range of 0–60 ppm [27], and Fabry Perot fiber sensors face the issue of non-linearity [28]. 

The setup has demonstrated a good sensitivity for toluene and has potential to be used for detecting BTEX molecules. The setup can be applied for ultra-low and high-selective detection of BTEX molecule mixtures by coupling it with a pre-concentration unit with/without a GC-column for separation purposes.

## 4. Conclusions and Future Scope

In this study we have demonstrated a simple and sensitive deep UV absorption spectrophotometry for detection of air-borne toluene. A fiber-coupled deep UV-LED was employed as a light source, and a spectrometer was used as detector with a gas cell in between. 3D printed opto-fluidics connectors were designed to integrate the gas flow with a UV source and detector. A glass HCW with aluminium coating and an aluminium HCW were tested as a gas cell. The setup was tested for different toluene concentrations (10–100 ppm), and a linear relationship was observed with sensitivities of 0.20 mA·U/ppm and 0.32 mA·U/ppm for glass HCW and aluminium HCW, respectively, at 260 nm. The limits of detection of 8.15 ppm and 12.45 ppm were calculated for glass HCW and aluminium HCW, respectively. The sensitivity and selectivity of the setup can be improved by coupling it with a pre-concentration unit and a micro GC column, respectively. This study provides a guide for the design of aluminium-based HCWs for UV spectrophotometry and can be applied to detect a number of molecules which show UV absorption for example, ozone, benzene, xylenes, NO_2_ and SO_2_.

## Figures and Tables

**Table 1:** Comparison of aluminium and glass HCWs for toluene detection.

HCW	Length (cm)	Wavelength (nm)	Sensitivity (mA·U·ppm^−1^)	Sensitivity/Length (mA·U·ppm^−1^·cm^−1^)
Aluminium	50	260.34	0.32	0.00640
		263.76	0.23	0.00460
		267.18	0.30	0.00600
Glass	34	260.34	0.20	0.00588
		263.76	0.15	0.00441
		267.18	0.19	0.00559
